# Flow Cytometric Detection of p38 MAPK Phosphorylation and Intracellular Cytokine Expression in Peripheral Blood Subpopulations from Patients with Autoimmune Rheumatic Diseases

**DOI:** 10.1155/2014/671431

**Published:** 2014-02-03

**Authors:** Athanasios Mavropoulos, Dimitrios P. Bogdanos, Christos Liaskos, Timoklia Orfanidou, Theodora Simopoulou, Efterpi Zafiriou, Lazaros I. Sakkas, Eirini I. Rigopoulou

**Affiliations:** ^1^Department of Rheumatology, Faculty of Medicine, School of Health Sciences, University of Thessaly, Biopolis, 41110 Larissa, Greece; ^2^Cellular Immunotherapy and Molecular Immunodiagnostics, Biomedical Section, Centre for Research & Technology Hellas (CE.R.T.H.)/Institute for Research and Technology-Thessaly (I.RE.TE.TH), 41222 Larissa, Greece; ^3^Division of Transplantation Immunology and Mucosal Biology, Institute of Liver Studies, King's College London School of Medicine King's College Hospital, London SE5 9RS, UK; ^4^Department of Dermatology, School of Health Sciences, Faculty of Medicine, University of Thessaly, Biopolis, 41110 Larissa, Greece; ^5^Department of Internal Medicine, School of Health Sciences, Faculty of Medicine, University of Thessaly, Biopolis, 41110 Larissa, Greece

## Abstract

Flow cytometric analysis of p38 mitogen-activated protein kinase (p38 MAPK) signaling cascade is optimally achieved by methanol permeabilization protocols. Such protocols suffer from the difficulties to accurately detect intracellular cytokines and surface epitopes of infrequent cell subpopulations, which are removed by methanol. To overcome these limitations, we have modified methanol-based phosphoflow protocols using several commercially available antibody clones suitable for surface antigens, intracellular cytokines, and p38 MAPK. These included markers of B cells (CD19, CD20, and CD22), T cells (CD3, CD4, and CD8), NK (CD56 and CD7), and dendritic cells (CD11c). We have also tested surface markers of costimulatory molecules, such as CD27. We have successfully determined simultaneous expression of IFN-**γ**, as well as IL-10, and phosphorylated p38 in cell subsets. The optimized phosphoflow protocol has also been successfully applied in peripheral blood mononuclear cells or purified cell subpopulations from patients with various autoimmune diseases. In conclusion, our refined phosphoflow cytometric approach allows simultaneous detection of p38 MAPK activity and intracellular cytokine expression and could be used as an important tool to study signaling cascades in autoimmunity.

## 1. Introduction

Regulation of cytokine gene expression in human lymphocyte cell subpopulations is tightly controlled by a dynamic interaction between harmonic and antagonistic signaling pathways [1–3]. The dissection of these molecular networks that are often dysregulated in autoimmune diseases is a major target of ongoing research [4–8].

Despite several technological advancements in molecular immunobiology, the *in vitro* study of rare cell populations, such as NK and NKT cells, has been always difficult, if not prohibitory. Multicolor flow cytometry is one of the advancements that have positively transformed translational research [9, 10]. This technology, initially used for the analysis of intracellular cytokine expression, is nowadays extended to involve recognition of phosphoepitopes as determinants of intracellular kinase activity within each single cell [11–13]. Thus, it serves as a viable alternative to traditional immunoblotting or kinase assays that require large numbers of homogenous cells [14, 15].

p38 MAPK signaling pathway regulates the expression of cytokines and chemokines by monocytes, NK, NKT, T, and B lymphocytes and plays an important role in the induction of autoimmunity [16–18]. Hence, we and others exploited phosphospecific flow cytometry (phosphoflow) protocols for the investigation of p38 activity in peripheral blood mononuclear cells (PBMCs) [19–22]. Successful detection of phosphorylated p38 (p-p38) MAPK in cell subpopulations is optimally achieved by methanol permeabilization. However, methanol may remove surface epitopes [23], making the accurate determination of rare cell subsets problematic, especially within unfractionated PBMCs [3, 22]. On the other hand, saponin, which is preferentially used for the detection of surface epitopes and intracellular cytokine expression, does not allow proper staining and antibody access to intracellular phosphospecific targets [24]. To overcome these problems, we have modified methanol-based phosphoflow protocols using several commercially available antibody clones suitable for surface staining, intracellular cytokine expression, and p-p38 labelling [19]. Thus, we have previously shown that our optimized p-p38 protocol allows accurate phenotypic discrimination of rare human NK and NKT cell subpopulations within unfractioned PBMCs from healthy blood donors, as well as proper IFN-*γ* expression [19, 20].

The first aim of the present study was to exploit this technology in order to allow proper determination of additional cell surface markers. These included the CD19, CD20, and CD22 markers of B cells, as well as the CD3, CD4, and CD8 T cell markers and the CD7, a complementary NK cell identification marker. We have also tested surface markers of costimulatory molecules, such as the T cell co-stimulatory molecule CD27, and activation markers, such as CD11c mostly expressed not only by human dendritic cells but also by NKs. The second aim of the present work was to determine whether it is possible to detect IL-10 and IFN-*γ* in cells with p-p38 MAPK. Finally, to further examine whether or not our phosphoflow approach can be applied for the study of autoimmunity, we have analyzed PBMCs or purified cell subpopulations isolated from patients with various autoimmune diseases and present these data herein.

## 2. Materials and Methods

### 2.1. Cell Separation and Purification

Peripheral blood (PB) samples (20 to 40 mL) were obtained by venipuncture from 12 healthy volunteer staff members (median age 35 years, range 21–53 years, 7 female) and 23 patients with various autoimmune diseases. Patients were recruited from the Outpatient and Inpatient Clinics of the Rheumatology Department, University General Hospital of Larissa, and included 7 patients with systemic sclerosis (SSc), 6 with Sjögren's syndrome (SjS), 4 with psoriatic arthritis (PsA), 4 with psoriasis, 1 with rheumatoid arthritis (RA), and 1 with Hashimoto's thyroiditis (HT). A written consent was obtained from all donors. The protocol was approved by the Local Ethic Committee of the University General Hospital of Larissa, University of Thessaly.

PB samples were collected in preservative-free heparin tubes (10 U/mL) and aliquots were layered onto an equal volume of Ficoll-Hypaque density gradient solution (Amersham Pharmacia Biotech Ltd., Little Chalfont, UK) and centrifuged at 300 ×g at 20°C. The mononuclear cells were collected and washed twice with serum-free RPMI-1640 (Invitrogen Life Technologies, Paisley, UK). Cell viability, determined by trypan blue exclusion, exceeded 97%. Isolated PBMCs were aliquoted into separate cryogenic vials (Corning, Sigma-Aldrich, St Louis, MO), kept at −80°C for one day and then transferred and stored in the vapour phase of liquid nitrogen. For signaling analysis, an individual cryotube (containing approximately 0.5 to 1 × 10^7^ PBMCs) was removed from liquid nitrogen and thawed in a 37°C water bath. Cells were washed with serum-free RPMI-1640 to remove the freeze medium, counted in 0.2% (w/v) trypan blue to ensure more than 95% cell viability, pelleted, and resuspended at 10^6^ cells/mL in RPMI culture medium supplemented with L-glutamine and 5% heat-inactivated fetal bovine serum (FBS). PBMCs were seeded in 24-well plates and allowed to rest at 37°C in a CO_2_ incubator for at least 1 h before stimulation.

### 2.2. Magnetic Cell Separation and Enrichment

Cells were separated using CD56 Microbeads (Miltenyi Biotec Inc.) and a MiniMACS separation column used according to the manufacturer's instructions. Purity was (>95%) as determined by FACS analysis.

### 2.3. Stimulation, Fixation, and Permeabilization Methodology

IL-12 (20 ng/mL), IL-18 (25 ng/mL) from R and D Systems Inc (Abington, UK) and phorbol 12-myristate 13-acetate (PMA) (50 ng/mL) and Ionomycin (1 *μ*M) (Sigma-Aldrich) were used to induce p38 MAPK phosphorylation in PBMCs. After stimulation, cells were kept in a 37°C CO_2_ incubator to allow signal transduction and phosphorylation. A parallel culture with unstimulated cells was used to determine basal levels of phosphorylation. Paraformaldehyde (10%, 200 *μ*L), prewarmed at 37°C, was added to the culture medium to give a final concentration of 2% and cells were incubated at 37°C for 15 min for fixation and detection of activated p38 MAPK. For intracellular cytokine detection, cells were treated with brefeldin A (GolgiPlug, BD Biosciences, Franklin Lakes, NJ) during stimulation to block cytokine secretion.

For cell permeabilization, two methods were employed. One was based on methanol and the other was based on saponin. In the first method, bovine serum albumin (BSA) (Invitrogen), FBS, and sodium azide (Sigma-Aldrich) were used to make a washing buffer (2% BSA/1% FBS/0.1% sodium azide in Tris-Based Saline (TBS)). After washing, cells were permeabilized by slowly (2–5 secs) adding 1 mL of 75–80% (v/v) methanol (Fisher Scientific, Pittsburgh, PA) in TBS, while thoroughly vortexing at medium speed. Cells were incubated for at least 30 min on ice and then stored in methanol/TBS at −20°C until further use. Alternatively, cells were permeabilized using 100 *μ*L of 1% BSA/0.1% sodium azide/0.2% saponin in TBS for 30 min. Saponin-treated cells were washed in TBS/1% BSA/0.2% saponin, while the methanol treatment groups were washed in 2% BSA/0.1% sodium azide in TBS.

Saponin-based permeabilization is a reversible process and therefore antibody staining should be always performed in saponin containing buffers. In contrast, methanol fixes and permeabilizes cells permanently. Cytofix/cytoperm buffer (BD Biosciences Pharmingen, San Diego, CA) was also alternatively utilised for saponin-based staining.

### 2.4. Intracellular Phosphospecific Flow Cytometry

Paraformaldehyde-fixed, methanol-permeabilized cells were rehydrated for 30–60 min by the addition of 1 mL of TBS-based wash buffer, followed by gentle resuspension and centrifugation. After washing, cells were resuspended in 50–100 *μ*L of blocking reagent containing 10% (v/v) human plasma in TBS or with an FcR blocking reagent (Miltenyi Biotec, Bergisch Gladbach, Germany), followed by 15 min incubation at room temperature (RT) to allow blocking of Fc receptors. The cell pellet was washed twice, resuspended in 50 *μ*L 2% BSA/TBS (w/v), and incubated with fluorochrome-conjugated antibodies. The following monoclonal antibodies (MoAb) were used Alexa 647 dye (Molecular Probes, Invitrogen) and phycoerythrin (PE) conjugated phospho-p38 MAPK (T180/Y182); fluorescein isothiocyanate (FITC)-conjugated anti-CD3 (clone UCHT1); (FITC)-conjugated anti-CD7 (clone 4H9); (PE)-conjugated anti-CD11c (clone SHCL-3); (PE)-conjugated anti-CD19 (clone HIB19); (Alexa 488)-conjugated anti-CD20 (clone FB1); (PC5)-conjugated anti-CD22 (clone HIB22); (FITC)-conjugated anti-CD27 (clone MT271); FITC- and APC-conjugated anti-hIFN-*γ* (clone 4SB3); and PE-conjugated anti-IL-10 (clone JES3-9D7), all obtained from BD Pharmingen. PE- and PC5-conjugated anti-CD56 antibodies (clone N901) were obtained from Beckman Coulter Inc. (Buckinghamshire, UK). According to the manufacturer, clone N901 does not react with human monocytes and granulocytes.

Appropriate titration of antibodies was performed for optimal detection of surface and phosphoepitopes. We typically titrated all selected surface and intracellular antibody clones to cover for a standard of 0.5–1 × 10^6^ cells per point. For intracellular p-p38 detection, we used previously optimised stimulation conditions and the antibody concentrations that allowed for the maximum detection [3]. To confirm the specificity of the phospho-p38 antibody, flow cytometry experiments were performed in the presence of p38 phospho-T180/Y182 or nonphosphopeptide competitors (Abcam, Cambridge, UK) [3]. The specific antibody for p-p38 MAPK has been also validated by direct comparison with Western blotting. An aliquot of cells was used as autofluorescence control and a second aliquot was stained with the appropriate isotype control IgG antibody. Incubations were performed at room temperature for 1 h. Insignificant background staining was observed using appropriate control FITC-conjugated, PE-, PC5-, and APC- or Alexa 647-conjugated antibodies. Flow cytometric analysis was performed by a FACS Calibur (Becton Dickinson Mountain View, CA) using logarithmic amplification and a forward and side scatter-based gate for total lymphocyte populations. At least 10^5^ cells within the lymphocyte gate were collected for each sample. BD CellQuest software (BD Bioscience) was used for data acquisition and off-line analysis. For each of the gated populations the percentage and geometric mean fluorescence intensity (MFI) were analyzed.

All values were expressed as mean ± standard deviation (SD), except otherwise stated.

## 3. Results

### 3.1. Phenotypic Analysis of Surface Epitopes of Cell Subpopulations Using Methanol-Based Permeabilization Protocols in Healthy Individuals (Figure 1)

**Figure 1:**
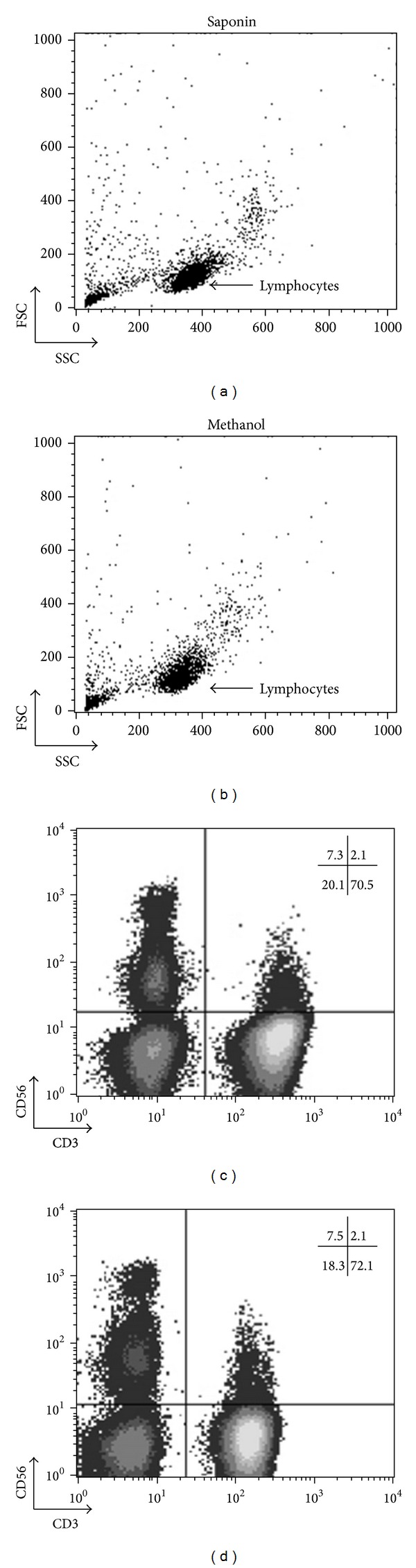
Comparable phenotypic analysis of peripheral blood NK, NKT, and T cells using saponin- or methanol-based permeabilization. Freshly isolated human PBMCs (1 × 10^6^)/mL were fixed using 2% paraformaldehyde, permeabilized in saponin- (left) or methanol- (right) based buffers, and stained with anti-CD3 and anti-CD56 MoAbs. Lymphocytes were gated on forward/side light scatter properties. Discrimination of CD56+CD3− (NK), CD56+CD3+ (NKT), and CD56−CD3+ (T) cells was comparable between the two methods.

Figure 1 illustrates representative results of freshly isolated PBMCs from healthy blood donors. PBMCs, fixed in 2% paraformaldehyde and permeabilized in saponin (left) or methanol-based buffers (right), were stained with anti-CD3 and anti-CD56 MoAbs. As shown, discrimination of CD56+CD3− (NK), CD56+CD3+ (NKT), and CD56−CD3+ (T) cells is comparable using either methodological approach. Depending on the integrity, the nature of the sample, and the MoAb, a 2–9% loss of surface labeling could be expected. This suggests that a thorough investigation of specific MoAbs should be done before analysis. Nevertheless, the methanol-based permeabilization protocol uniformly maintains the integrity of surface antigens, allowing us to easily discriminate the expression levels of cell subpopulations, such as CD56+ and CD3+.

### 3.2. Methanol-Based Permeabilization Permits Analysis of Cell Surface Markers, including Activation and Costimulatory Molecules (Figure 2)

**Figure 2:**
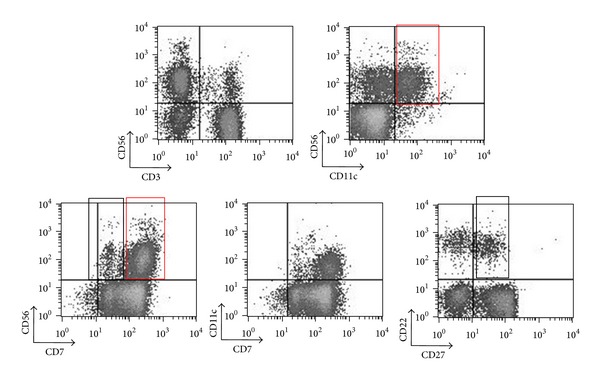
Surface staining of CD3, CD56, CD7, CD11c, CD20, CD22, and CD27 epitopes with methanol-based permeabilization. Freshly isolated human PBMCs from a healthy donor were fixed using 2% paraformaldehyde, permeabilized in methanol, and stained with MoAb clones against CD3, CD56, CD7, CD11c, CD22, and CD27. Lymphocytes were gated on forward/side light scatter properties. Successful discrimination of CD56+CD3−, CD56+CD3+, CD56−CD3+, CD56+CD11c+, CD56+CD11c−, CD56+CD7+, CD22+CD27+, and CD22+CD27− cells was demonstrated. A clear distinction between high CD56 and medium/low CD56 as well as high CD7 and medium/low CD7 was noted (first row, right plot and second row, left plot, resp.).

In search for additional surface epitopes that will expand our capability to correctly characterise PBMCs subpopulations, we tested several commercial MoAbs against standard phenotypic markers using both permeabilization methods. Figure 2 illustrates a representative panel of successfully stained surface epitopes in methanol-permeabilized PBMCs from a healthy individual. To better define the CD56+ subpopulation, we stained the cells using the CD7 marker [25], as coexpression of CD7 and CD56 differentiates NK cells from CD56+ monocyte/DC-like cells, which lack CD7 [26]. We detected moderate levels of CD7 expression (Figure 2, lower panel, left plot, black box) in a fraction of CD56 cells (13.4 ± 4.5%, 7 healthy controls), as previously described [27], indicating differences in the activation states of the NK cells [28]. The majority of CD56+ cells expressed CD7 at a high density (Figure 2 lower panel left plot, red box). Differences in the levels of co-expression of CD4 and CD8 surface antigens by peripheral blood CD3+ cells were also noted (data not shown).

We also stained with anti-CD11c MoAb to better define DCs (high CD11c expression) [29–31] or other cell populations, such as activated NKs (medium/low CD11c expression) [32–34]. CD11c expression was detected in approximately half of CD56+ cells also coexpressing CD7 (55 ± 5.6%, 7 healthy controls). A representative case is also shown in Figure 2. CD56+CD11c+ cells are illustrated in upper panel (right plot, red box). CD11c+ cells are also detected in purified human NK fractions [32], and phenotypic classification becomes more difficult as CD56+ DCs [26, 35, 36] and bitypic NK/DC cells have been described [37]. Although technical difficulties were described in various studies, we have noted that CD56, CD7, and CD11c stainings perform well (Figure 2, upper panel, right plot and Figure 2, lower panel, middle plot). Similar results were obtained in magnetically selected CD56+ cells (data not shown).

We stained for various B cell specific markers, such as CD19, CD20, CD22, and CD27 (an activation marker indicative of memory B cells) [38], which is also expressed in mature NKs [39, 40]. In the present study, 54.7% ± 5.6% (7 healthy controls) of CD22-expressing B cells also coexpressed CD27 (Figure 2, lower panel, right plot, black box). CD22+ B cells were CD20+CD19+ (saponin protocol, data not shown). The CD19 antibody clone did not stain satisfactorily under methanol-based permeabilization. Therefore, a CD20, CD22, and CD27 combination was preferred to better define the B-cell subpopulations within PBMCs (Figure 2, lower panel, right plot).

### 3.3. PBMCs Subpopulations Distinguished by Methanol-Based Protocol in Patients with Autoimmune Disorders (Figure 3)

**Figure 3:**
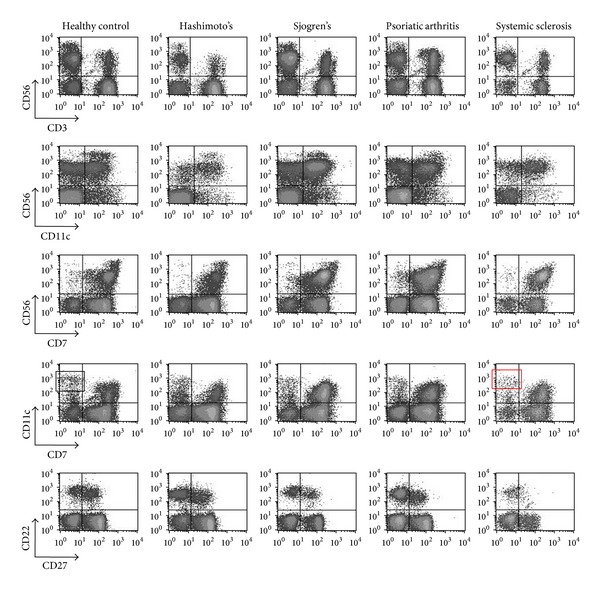
Surface epitope staining in methanol-permeabilized PBMCs of patients with autoimmune disorders. PBMCs from a representative healthy individual and patients with autoimmune disorders were fixed in 2% paraformaldehyde, permeabilized in methanol, and stained with MoAbs specific for CD3, CD56, CD7, CD11c, CD22, and CD27. Lymphocytes were gated on forward/side light scatter properties. Surface epitope staining was preserved in samples from healthy controls and autoimmune patients. The distinction of PBMCs subpopulations was clear.

In view of the profound differences regarding the integrity, quality, viability, and total number of cells between healthy controls and patients, we performed a series of experiments in patients with autoimmune disorders. A representative panel of a typical surface staining of PBMCs from patients with autoimmune diseases using methanol permeabilization is illustrated in Figure 3. This figure shows results obtained in individual patients with SjS, SSc, PsA, and HT as well as in a healthy control. In general, we could demonstrate that the methanol-based protocol allows a clear discrimination of surface markers, such as CD56/CD3 (first column), CD56/CD11c (second column), CD56/CD7 (third column), CD11c/CD7 (fourth column), and CD22/CD27 (fifth column). Subtle differences amongst patients and healthy controls are readily visible. For example, the SSc patient (lower panel, column 4, red box) is characterized by lower CD11c^high^CD7− (DCs) compared to the healthy control (upper panel, column 4, black box).

### 3.4. p-p38 MAPK Positive Subpopulations in PBMCs from Patients with Autoimmune Disorders (Figure 4)

**Figure 4:**
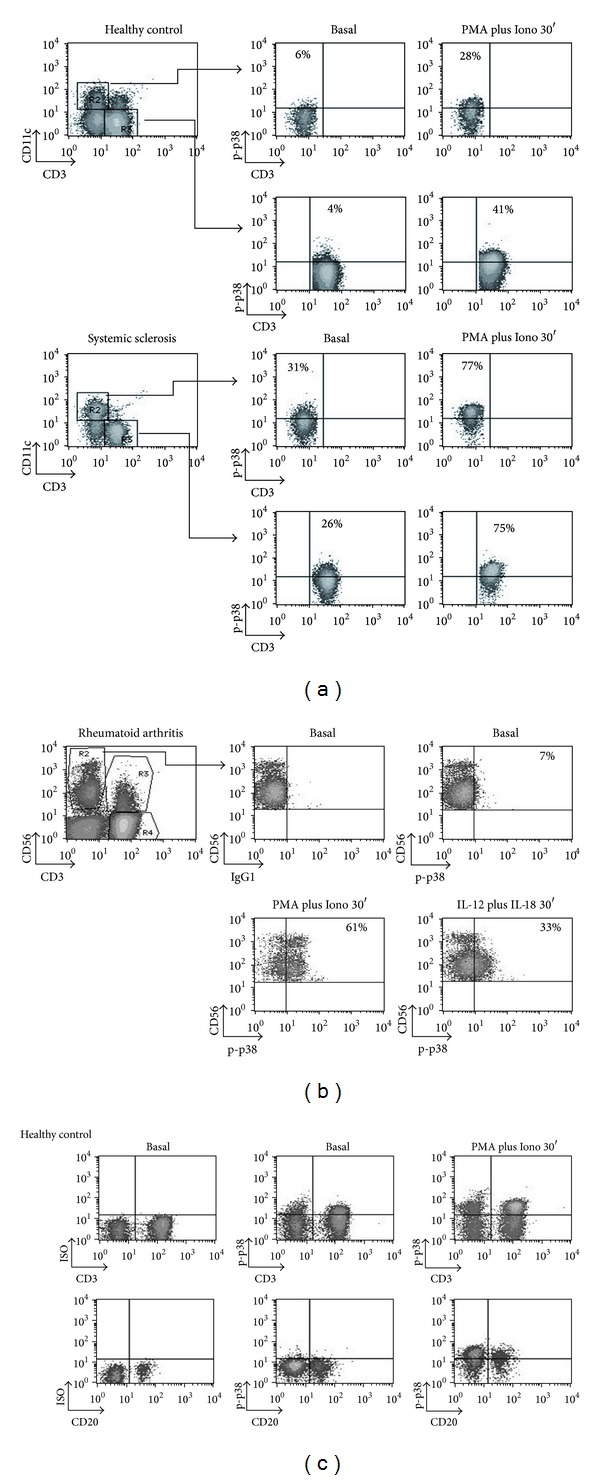
p-p38 detection in PBMCs subpopulations from normal controls and patients with autoimmune diseases. PBMCs from a representative healthy individual and patients with autoimmune disorders ((a), (b), and (c)) were left untreated or stimulated with PMA plus Ionomycin for 30 min and stained with MoAbs against CD3, CD56, CD11c, CD20, and p-p38. Cell subsets were subgated based on surface epitope staining and plotted for p-p38. (a) A representative comparison of CD11c+ p-p38+ and CD3+ p-p38+ cells between a healthy control and a patient with systemic sclerosis is shown. (b) PBMCs stimulated with PMA plus Ionomycin or IL-12 plus IL-18. Shown here are percentages of CD56+ CD3− p-p38+ cells from a RA patient using different stimuli (Gate R1). (c) Shown here are CD3+ p-p38+, CD3− p-p38+, CD20+ p-p38+, and CD20− p-p38+ cells after 30 min stimulation from another healthy donor.

The protocol allows p38 MAPK determination in cell subpopulations, such as CD11c^high^, which participate in the DC/T interactions of innate and adaptive immunity [29, 41]. p-p38 MAPK in a representative case of a patient with SSc and a normal control is illustrated in Figure 4(a). p-p38 MAPK was analyzed in CD11c+ and CD3+ cells stimulated with PMA plus Ionomycin for 30 min. The patient with SSc had higher p-p38 MAPK positive CD11c+ and CD3+ cells compared to the normal control. In Figure 4(b), a patient with RA was treated with PMA and Ionomycin or IL-12 plus IL-18 (for 30 min). Subgated CD56+ cell analysis showed clear p-p38 MAPK induction, allowing further analysis. A representative experiment showing p38 MAPK phosphorylation within CD3+ and CD20+ subpopulations from a healthy donor is shown in Figure 4(c).

### 3.5. Methanol-Based Permeabilization Allows Optimal Cytokine Expression Analysis (Figure 5)

**Figure 5:**
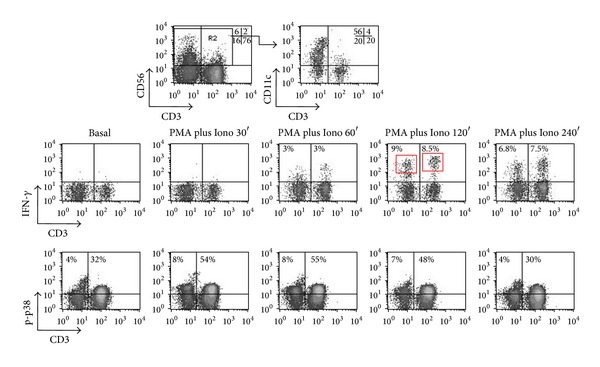
Optimal cytokine expression and p-p38 detection within PBMCs subsets permeabilized with methanol. PBMCs were stimulated with PMA plus Ionomyci at various time points, permeabilized in methanol, and stained with MoAbs against CD3, CD56, CD11c, p-p38, and IFN-*γ*. CD56+ cells were subgated and the percentage of CD11c+ within CD56+ cells is indicated. Gated CD56+ cells were plotted for CD3 versus IFN-*γ* and CD3 versus p-p38. As it can be seen, IFN-*γ* expression is detectable between 60′ and 240′, reaching its peak at 120′, while maximal p-p38 activity is better visualized at 30′. NKT cells express more IFN-*γ* than NK as indicated by the measured percentages and mean fluorescence intensity of IFN-*γ*+ NKT cells (see text for more details).

The protocol applicability for intracellular cytokine expression has been validated. We have noted that stimulation with PMA and Ionomycin can optimally promote simultaneous p-p38 MAPK and IFN-*γ* expression at the same time point (Figure 5). This cannot be seen by IL-12 and IL-18, as p-p38 is achieved within minutes, while intracellular IFN-*γ* is detectable at 120–240 min, as previously described [3]. Based on our experiments (Figure 5) and previously published data [23], PMA and Ionomycin allow prolonged and strong p38 MAPK activation and promote the earliest possible cytokine expression in PBMCs. These findings demonstrate that the combination of PMA and Ionomycin is an ideal *in vitro* stimulus for these types of experiments. Figure 5 illustrates an example of kinetics of IFN-*γ* protein expression over time (0, 0.5, 1, 2, and 4 h) in CD56+ gated cell subpopulations of PBMCs after stimulation with PMA and Ionomycin. In this representative example of a healthy blood donor, NKT cells have higher mean fluorescence intensity (MFI) of IFN-*γ* expression than NK cells (Figure 5 middle row, plot 4, red boxes). This is best seen at 120 min following PMA and Ionomycin treatment, with NKT cells having 2.5 times higher MFI of IFN-*γ* expression than NK cells (365 for NKT cells versus 164 for NK cells).

### 3.6. Simultaneous Cytokine and p-p38 MAPK Expression in PBMCs Cell Subpopulations (Figure 6)

**Figure 6:**
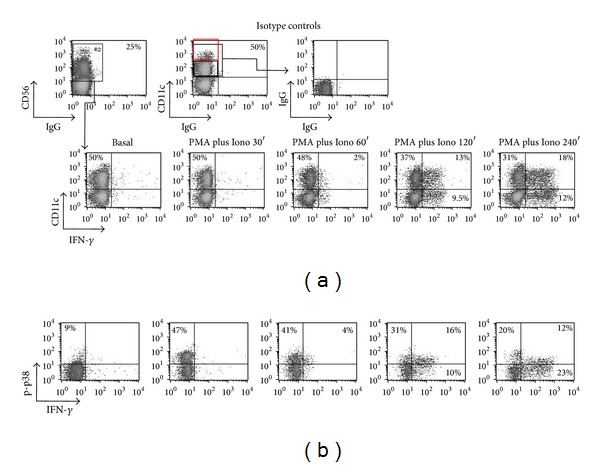
Simultaneous detection of IFN-*γ* and p-p38 MAPK expression in CD56+ CD11c+ cell populations. PBMCs were stained with MoAbs against CD56, CD11c, p-p38, and IFN-*γ*. CD56+ cells were subgated and the percentage of CD11c+ within CD56+ cells is shown. (a) Gated CD56+ cells were plotted for CD11c versus IFN-*γ* in order to assess IFN-*γ* expression within CD11c+ cells. (b) Gated CD56+ CD11c+ cells were plotted for IFN-*γ* versus p-p38 to measure double positivity for p38 and IFN-*γ*. A significant expression of early IFN-*γ* is detected within the CD11c+ CD56+ cell subpopulation.

The previous set of data showed the ability of p38 MAPK phosphorylation and expression of IFN-*γ* detection in response to PMA and Ionomycin. The results seen in Figure 6 illustrate four-parametric analysis with simultaneous detection of intracellular cytokine (IFN-*γ*) and p-p38. PBMCs from a representative donor were treated with PMA and Ionomycin or IL-12 and IL-18 and four markers were investigated CD56, CD11c, p-p38 MAPK, and IFN-*γ*. The integrity of surface staining was maintained allowing for clear distinction between bright (Figure 6, upper panel, right plot, red box) and dim CD11c expression (black box). This distinction has originally been reported in murine NKs (DX5+); a rapid IFN-*γ* expression has also been noted in murine CD11c+ (NK1.1+) cells [32, 33, 42–44]. Our findings (Figure 6) show that these cells (CD11c+) express IFN-*γ* and p-p38 MAPK.

### 3.7. Time-Dependent Intracellular Cytokine Expression in p-p38+ Cells (Figure 7)

**Figure 7:**
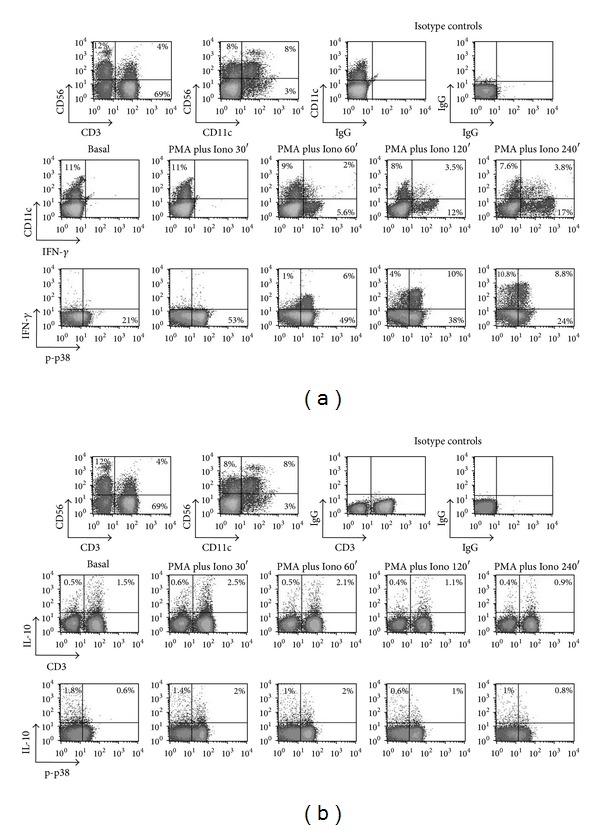
Simultaneous detection of IFN-*γ*, IL-10, and p-p38 MAPK expression in PBMCs cell subpopulations. (a) shows the plots for CD11c versus IFN-*γ* and IFN-*γ* versus p-p38. (b) shows the plots for CD3 versus IL-10 and IL-10 versus p-p38. In addition to IFN-*γ*, IL-10 can be detected under harsh methanol permeabilization conditions. IL-10 expression precedes that of IFN-*γ* and can be seen in both CD3+ and CD3− cells.

In addition to IFN-*γ*, we assessed intracellular cytokine expression of IL-10, an anti-inflammatory cytokine. Figure 7 depicts a representative experiment of time-dependent IL-10 expression in PBMCs from a healthy individual stimulated with PMA and Ionomycin. An analysis was also carried out for CD56, CD3, CD11c, p-p38, and IFN-*γ* (Figure 7(a)). As shown, IL-10 is expressed 30 min to 60 min after stimulation, whereas IFN-*γ* is better detected at later time points (120 min, and 240 min) (Figure 7(b)).

## 4. Discussion

In the present study, we convincingly show that our refined phosphoflow protocol allows simultaneous measurement of p38 MAPK phosphorylation and intracellular cytokine expression in various PBMCs subpopulations. This approach has revealed differences amongst patients with autoimmune diseases and normal controls and could be used as an important tool to study signaling cascades in autoimmunity.

Phosphoflow protocols were unable to meet research needs for the characterization of infrequent PBMCs subpopulations, such as high and low CD56+, CD11c+ CD7+ NK cells, and CD56+ DCs, because of insufficient detection. Thus, several cell surface epitopes are removed by methanol fixation, as they are maintained on the cells loosely and can be stripped off the cell surface. Furthermore, most epitopes on several surface proteins are detectable only after extensive rehydration. This can explain previous findings showing that CD56 overexpressing cells are virtually undetectable in PBMCs when 90% methanol treatment was used [23]. We demonstrate that moderate methanol concentration (75–80% (v/v)) and subsequent rehydration are sufficient for optimal phenotypic analysis. Antigen accessibility, stability of the phosphoepitope, and antibody suitability for staining inside fixed and permeabilized cells should be considered [24, 45]. For example, a CD19 MoAb did not work well in our hands. Phosphoflow approaches also have limitations in the determination of intracellular cytokines. However, moderate methanol concentration overcomes these limitations and permits accurate determination of cytokines, comparable to saponin protocols [45, 46].

The molecular mechanisms that promote NK production of proinflammatory, instead of anti-inflammatory, cytokines are incompletely understood, but some clues have been obtained by the investigation of p38 MAPK [47, 48]. Thus, we have previously demonstrated that the p38 MAPK pathway regulates the induction of IFN-*γ* in IL-12- and IL-18-stimulated human NK cells by stabilizing IFN-*γ* mRNA transcripts [3]. This implies that, as described in other cell types such as monocytes [49, 50], a mechanism of posttranscriptional gene expression directed by p38 MAPK may exist in NKs. Posttranscriptional regulation of IL-10 gene expression has been described for NK cells but it is unclear whether this is due to p38 MAPK signaling [51–53]. On the other hand, as for IFN-*γ*, p38 inhibitors can block expression of IL-10 in murine NKs [54, 55].

We extended our previous findings demonstrating accurate determination of p-p38 and IFN-*γ* in NK and NKT cell subpopulations [19, 20] to include several other surface epitopes. For example, we successfully applied anti-CD22 and anti-CD27 MoAbs for the distinction of memory and naïve B cells [38]. This approach has applications in the study of autoimmune diseases; our preliminary experiments showed increased percentages of p-p38+ cells amongst B cells in autoimmune rheumatic disorders. Additional NK markers, such as CD7 and CD11c (also expressed in DCs), were also successfully determined. Simultaneous detection of these markers allows delineation of p-p38 MAPK pathway in distinct NK, DC, and NKDC populations [56–59], in relation to cytokine expression (IFN-*γ*, IL-10) [51, 60]. This methanol-based approach could be applied for the study of innate immune responses in autoimmune disorders [2, 61]. For example, our data show that CD56+ CD7+ cells expressing CD11c can phosphorylate p38 MAPK and express IFN-*γ*. Several studies have also demonstrated that murine B220+ CD11c+ NK1.1+ populations, originally described as dendritic cells or NK/DCs with both killing and antigen presenting cell capabilities, may actually represent activated NK cells [62–67]. Meticulous assessment of these features may provide a better understanding of the induction of autoimmunity.

In summary, we provide a protocol that allows successful discrimination of several PBMCs subpopulations. This permits detection of intracellular p38 MAPK without sacrificing a simultaneous detection of intracellular cytokines. Other kinases, such as ERK1/2, transcription factors, such as STAT4, STAT5, and cytokines may also be analyzed [68–70]. It should be reminded that the aim of the present study was not to determine differences of p-p38 MAPK and cytokine expression within cell subpopulations among patients with autoimmune disorders. In fact, our goal was to assess whether our refined p-p38 MAPK protocol can be used as a tool to study pathogenetic mechanisms involved in autoimmunity [17, 71, 72].

## Abbreviations

IFN-*γ*: Interferon-*γ*
IL-10: Interleukin-10
HT: Hashimoto's thyroiditis
MAPK: Mitogen-activated protein kinase
MFI: Mean fluorescence intensity
MoAb: Monoclonal antibodies
PB: Peripheral blood
PBMC: Peripheral blood mononuclear cells
p-p38: Phospho-p38
PsA: Psoriatic arthritis
RA: Rheumatoid arthritis
SjS: Sjögren's syndrome
SSc: Systemic sclerosis.
